# The prevalence and impact of childhood sexual abuse on HIV-risk behaviors among men who have sex with men (MSM) in India

**DOI:** 10.1186/s12889-016-3446-6

**Published:** 2016-08-12

**Authors:** Cecilia Tomori, Allison M. McFall, Aylur K. Srikrishnan, Shruti H. Mehta, Nymisha Nimmagadda, Santhanam Anand, Canjeevaram K. Vasudevan, Suniti Solomon, Sunil S. Solomon, David D. Celentano

**Affiliations:** 1 Department of Epidemiology, Johns Hopkins Bloomberg School of Public Health, 615 N. Wolfe Street, E6648, Baltimore, MD 21205 USA; 2 YR Gaitonde Centre for AIDS Research and Education, Chennai, India; 3 Johns Hopkins Krieger School of Arts and Sciences, Baltimore, MD USA; 4 Department of Medicine, Johns Hopkins School of Medicine, Baltimore, MD USA

**Keywords:** Childhood sexual abuse, HIV, Risk-behaviors, Gender nonconformity, Men who have sex with men, India

## Abstract

**Background:**

Childhood sexual abuse (CSA) is a significant global public health problem, which is associated with negative psychosocial outcomes and high-risk sexual behaviors in adults. Men who have sex with men (MSM) often report higher prevalence of CSA history than the general population, and CSA may play a key role in MSM’s greater vulnerability to HIV.

**Methods:**

This study examined the prevalence of CSA history and its impact on the number of *recent* HIV-related risk behaviors (unprotected anal intercourse, high number of male and female sexual partners, alcohol use, drug use, and sex work in prior 6 months) and *lifetime* risk behaviors and experiences (high number of lifetime male and female sexual partners, early sexual debut, injection drug use, sex work, and intimate partner violence) among 11,788 adult MSM recruited via respondent driven sampling across 12 sites in India, with additional insights from thematic analysis of qualitative research with 363 MSM from 15 sites.

**Results:**

Nearly a quarter (22.4 %) of participants experienced CSA, with substantially higher prevalence of CSA in the South and among *kothis* (feminine sexual identity). Qualitative findings revealed that older, trusted men may target young and, especially, gender nonconforming boys, and perpetrators’ social position facilitates nondisclosure. CSA may also initiate further same-sex encounters, including sex work. In multivariable analysis, MSM who experienced CSA had 21 % higher rate of *recent* (adjusted rate ratio [aRR = 1.21], 95 % confidence interval [CI]: 1.14–1.28), and 2.0 times higher *lifetime* (aRR = 2.04, 95 % CI: 1.75–2.38) HIV-related behaviors/experiences compared with those who did not.

**Conclusion:**

This large, mixed-methods study found high overall prevalence of CSA among MSM (22.4 %), with substantially higher prevalence among MSM residing in the South and among more feminine sexual identities. Qualitative findings highlighted boys’ vulnerabilities to CSA, especially gender nonconformity, and CSA’s role in further sexual encounters, including sex work. Additionally, CSA was associated with an elevated rate of *recent*, and an even higher rate of *lifetime* HIV-related risk factors. Our results suggest an acute need for the development of CSA prevention interventions and the integration of mental health services for MSM with histories of CSA as part of HIV-prevention efforts.

**Electronic supplementary material:**

The online version of this article (doi:10.1186/s12889-016-3446-6) contains supplementary material, which is available to authorized users.

## Background

Childhood sexual abuse (CSA) is a significant global health problem, with numerous well-established adverse effects [1]. According to the 2006 World Report on Violence Against Children, 223 million children experienced CSA but underreporting, lack of safe and confidential assessment, and inconsistent criteria for measurement of CSA across settings makes establishing the prevalence of CSA challenging [1]. CSA has been identified as an important predictor of high risk sexual behavior later in life for both men and women, including early sexual debut, unprotected sex, multiple partners, and engagement in transactional sex, which increase the risk of HIV and other sexually transmitted infections (STIs) [2]. Numerous studies have documented that men who have sex with men (MSM) reported high prevalence of CSA history, with estimates ranging from 15.1 % in China [3] to 39.7 % in the U.S. [4] and 42 % in Latin America [5]. U.S. studies have found that MSM were more often victims of CSA compared to the general population [6, 7]. History of CSA among MSM is associated with a number of psychosocial concerns, including alcohol and drug use, depression, posttraumatic stress disorder (PTSD), suicidality, intimate partner violence, and forced sex [6–9]. Consequently, CSA has also been conceptualized as part of a syndemic of co-occurring psychosocial factors that enhance sexual minorities’ vulnerability to HIV [10].

The implications of CSA for HIV risk behaviors and HIV infection are profound. In a large study of 4295 MSM from 6 U.S. cities recruited for an HIV intervention (EXPLORE study), nearly forty percent had a history of CSA [4]. These men had significantly higher odds of engaging in unprotected anal sex (UAI) and in serodiscordant UAI, and were at increased risk for becoming infected with HIV during the course of the study follow-up period. In a recent analysis from the EXPLORE study, five syndemic conditions, including CSA, depression, heavy alcohol use, stimulant use and polydrug use, were found to enhance the likelihood of HIV seroconversion, in direct proportion to the number of syndemic conditions experienced. Although comparatively less is known about CSA and other syndemic conditions among MSM from low and middle income countries (LMIC), there is growing evidence that CSA plays a crucial role in these syndemics as well [3, 5, 11].

While global estimates have identified comparatively lower CSA prevalence in Asia [12], the prevalence of CSA in India is exceptionally high [13, 14]. In the largest study to date across 13 states (*n* = 12,447), over half of children (53 %) reported CSA, and a fifth (21 %) experienced more severe forms of abuse. The prevalence of both CSA overall and severe CSA was higher for boys than girls (53 and 47 %, and 23 and 19 %, respectively) [15]. The strikingly high prevalence of CSA among boys is particularly unusual, and stands in contrast with the majority of global trends [1, 12, 16]. The majority of perpetrators were known to the abused children, and many of them were extended family members. Most children did not report the abuse. Although the findings of this study cannot be generalized, smaller studies from India have also reported very high prevalence of CSA [13, 17, 18]. High prevalence of poverty and lack of education, the perception of children as governed by adults’ authority, the lack of sexual education, and devaluation of girl children have been cited as reasons for this high prevalence [13, 17, 18]. The secrecy surrounding abuse because of the damaging effects on the entire family’s reputation, and mistrust of the police are key reasons for difficulty in disclosure of the abuse.

To date, the literature in India has emphasized CSA targeting girls, leaving the vulnerabilities of boys largely unexplored. Moreover, there is no data on the prevalence of CSA among MSM compared with the general population. Qualitative studies, however, suggest that early childhood sexual experiences are frequently reported among MSM [19–21], with many instances explicitly construed in terms of forced sex and abuse [19], while others may not be described in these terms but reflect substantial power differences between the child and the person who approaches the child with sexual intentions [20].

MSM in India have a 12–14 times higher prevalence of HIV than other men [22]. Homosexuality is criminalized, and men are expected to conform to cultural norms of masculinity in behavior and appearance, heterosexual marriage, and the production of children [23–28]. At the same time, male-to-male sexual contact is common, although it may not be considered homosexuality [23, 27, 29]. There is considerable diversity in sexual identities among MSM, ranging from no explicit identity to the most frequently used *kothi* (feminine, predominately practicing receptive anal intercourse), *panthi* or *girya* (masculine, predominately practicing insertive anal intercourse), *double decker* or *DD* (masculine or feminine, practicing either sexual role), besides *bisexual* and *gay* identities that tend to be associated with higher social classes [26, 30–32]. Previous studies have documented that MSM engage in high-risk sexual behavior and experience poor psychosocial health [21, 33–40], but the role of CSA remains unexplored in these studies. This large multi-site mixed methods study across 15 sites in 5 states and a Union Territory examines the prevalence and social context of CSA among MSM. Building on the growing body of research that recognizes the role of multiple co-occurring factors that contribute to HIV-vulnerabilities, the study investigates the association of CSA with the cumulative number of reported HIV-related risk behaviors and experiences among MSM.

## Methods

Data for this research are drawn from a cluster-randomized HIV-prevention trial among MSM in India (ClinicalTrials.gov Identifier: NCT01686750) [40, 41]. The qualitative component of this research is drawn from the formative research phase of this trial, while the quantitative component originates from the baseline data collection for the trial.

### Qualitative data collection and analysis

#### Study design and procedures

As part of the formative qualitative research for the cluster-randomized trial, thirty-one focus group discussions (FGDs) and 121 in-depth interviews (IDIs) were conducted by trained interviewers with 363 MSM from 12 study sites and 3 additional sites (Chittoor, Andhra Pradesh; Tumkur, Karnataka; Trichy, Tamil Nadu) in local languages. The distribution of participants across sites and FGDs/IDIs has been published elsewhere [42]. Participants were identified by local NGOs who provide services for MSM and by peers based on their knowledge about and/or involvement in outreach work with MSM. FGDs and IDIs addressed a wide-range of topics related to the experiences of MSM in their communities and the availability and accessibility of HIV-related services for MSM, using open-ended questions whenever possible. FGDs, and especially IDIs, explored participants’ process by which they came to have sex with men and their sexual identities as well as their present-day sexual activities. Participants were compensated for their time.

#### Qualitative data analysis

FGDs and IDIs were transcribed, translated into English and entered into Atlas. TI qualitative software (version 7.5, Scientific Software Development GmbH, Eden Prarie, MN). Transcripts were read multiple times by the lead qualitative researcher (CT) [43], and emergent themes were identified following the principles of grounded theory using open coding and the constant comparison method [44, 45]. These themes were refined, and used to develop a preliminary codebook that was elaborated based on discussion with the study team [44, 45]. For this analysis, the emergent theme of CSA was then further explored by an additional round of coding carried out by two coders (CT and NN), which yielded a final coding scheme with a codebook that was applied to the transcripts. Any coding differences were resolved through discussion and finalized by the lead qualitative researcher (CT). Quotations were selected to illustrate each CSA-related subtheme, with the state, site, and sexual identity of the participants. Below, we summarize the qualitative results that specifically address CSA-related findings. Finally, we draw on qualitative findings to yield possible interpretations of the results from the quantitative analyses.

### Quantitative data collection and analyses

#### Study design

**Fig. 1:**
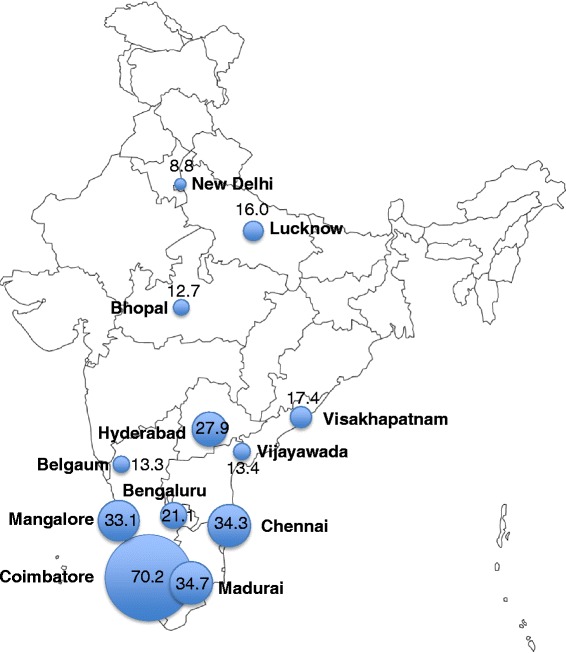
Prevalence of CSA by site. 95 % confidence intervals: Bengaluru 17.6–24.6 %, Belgaum 10.6–16.0 %, Bhopal 9.9–15.5 %, Chennai 29.7–38.9 %, Coimbatore 65.7–74.6 %, New Delhi 6.2–11.4 %, Hyderabad 24.1–31.6 %, Lucknow 13.0–19.0 %, Madurai 30.0–39.5 %, Mangalore 27.9–38.4 %, Vijayawada 10.5–16.3 %, Visakhapatnam 12.5–22.4 %.

The quantitative study was conducted in 12 cities from five states and one Union Territory of India as the baseline assessment of the cluster-randomized trial referenced above (Fig. 1) [40, 41]. Study participant eligibility criteria included: (1) age ≥ 18 years; (2) self-identify as male; (3) report oral or anal sex with another man in the prior 12 months; (4) provide informed consent; and (5) possess a valid respondent driven sampling (RDS) referral coupon (except for “seeds”, individuals identified in the qualitative phase as well-connected in the MSM communities, who initiated recruitment). Participants who self-identified as female or transgender (*hijra*) were excluded.

#### Study procedures

Detailed study procedures for the baseline assessment have been published elsewhere [40]. Briefly, the study population was recruited utilizing respondent-driven sampling (RDS), a chain-referral strategy for recruiting hard-to-reach participants whereby the resulting sample is considered representative of the target population [41, 46, 47]. We initiated recruitment at each of the 12 sites with two or three ‘seeds’ as described above. Target recruitment was 1000 per site. After verbal consent, participants completed an interviewer-administered survey and underwent rapid HIV testing and pre- and post-test counseling on-site. Each participant who completed the study was given two coupons to recruit other individuals from his network. Participants were reimbursed for participating in the study and for each eligible participant they recruited. Coupons were bar-coded to track recruitment chains and imprinted with a holographic image to hinder duplication. Enrollment was stopped when each site reached the target sample size.

#### Assessment of CSA and HIV-related risk behaviors and experiences

History of CSA was assessed within the interviewer-administered electronic survey by asking each participant whether he “experienced any unwanted sexual experiences (i.e., sexual touching or sexual intercourse, either oral or anal) when he was growing up (before 16 years old).” For this analysis, we focused on the association of CSA with behaviors and experiences that are well established in the biomedical literature as risk factors for HIV infection [40, 48–53]. Specifically, *recent* behaviors of interest were unprotected anal intercourse (UAI) (receptive or insertive), high number of male and female sexual partners, hazardous alcohol use or alcohol dependence (measured using the AUDIT instrument [54, 55]), injection drug use, non-injection drug use, and sex work. With the exception of alcohol use and dependence, which reflects alcohol consumption and alcohol-related problems within the prior 2 weeks, all other recent behaviors were within 6 months prior to the interview. *Lifetime* behaviors and experiences of interest were high number of lifetime male and female sexual partners, early sexual debut (before 15 years old), as well as reporting ever injection drug use, sex work, and intimate partner violence (IPV). Not all variables of interest were available in both *recent* and *lifetime* timeframes. IPV was measured only as a lifetime variable, while alcohol and non-injection drug use were only measured as recent behaviors. For both *recent* and *lifetime*, a high number of male sexual partners was defined as being within the top quartile of the sample distribution and a high number of female partners was defined as being within the top decile. *Recent* and *lifetime* history of HIV-related behaviors and experiences were also collected by self-report via the interview-administered survey.

#### Statistical analyses

Site-level prevalence of CSA was estimated using the RDS-II estimator (Volz-Heckathorn estimator), which weights estimates for network size (i.e., the number of MSM in the city whom the participant saw in the prior 30 days) [46]. Population summary statistics for socio-demographic, behavioral, and psychosocial factors were estimated with a composite weight, which accounts for the relative population size of adult men 15–59 years of age in each city [56] (assuming a similar proportion of MSM across cities) in addition to the RDS-II weight. To statistically compare characteristics of those with and without experiences of CSA we used likelihood ratio tests and their associated *p*-values from multi-level logistic regression models with random-intercepts for each site. The likelihood ratio test tests whether the less restrictive model of CSA (i.e. with the predictor/characteristic of interest) fits the data significantly better than the more restrictive model (i.e. intercept-only model of CSA with no predictors) [57].

We explored the relationship of CSA with the cumulative number of HIV-related risk behaviors and experiences. A score was calculated as the sum of risk behaviors and experiences present for each participant; *recent* and *lifetime* scores were constructed separately with score ranges of 0 to 7 and 0 to 6, respectively. The association between CSA and the HIV risk scores were modeled using multi-level poisson regression models resulting in rate ratios (RR). Models included random-intercepts for each site (to account for clustering) and incorporated scaled RDS-II sampling weights. Sexual identity was hypothesized to be a confounder of the CSA-HIV risk score association, and therefore the final multivariable models were adjusted for sexual identity. Univariable multilevel logistic regressions were also run separately for each risk behavior/experience as an additional exploratory analysis with these results provided in the supplementary tables. Seeds were excluded from all analyses. Unweighted model results are provided in supplemental tables. All statistical analyses were performed using the RDS Analyst Software version 0.1 (http://hpmrg.org) and STATA version 13.0 (STATA Corp., College Station, Texas, USA).

## Results

### Qualitative results

#### Participant characteristics

Qualitative research participant characteristics have been described elsewhere [42]. Briefly, participants’ median age was 30 (IQR 25–35), 41 % were ever married to a woman, and 40.8 % were *kothi*, a third were *DD* (31.4 %), and the remaining were *panthi*/*girya* (15.2 %), *bisexual* (9.4 %), and *gay* (3.3 %).

#### Overview of early sexual experiences

Participants frequently discussed sexual encounters prior to the age of 16 with other boys and men across the sites as part of their discussions of sexual experiences and sexual identity formation in IDIs and in FGDs. Nearly all childhood sexual experiences were reported by *kothis,* with few by *DDs* and *panthis*. Few cases involved younger children (beginning with age 3), while most childhood sexual experiences took place later on, usually during just prior to or during adolescence. The majority of participants did not explicitly label these encounters as instances of abuse; instead participants often reported that a sexual experience took place with an older person in a matter-of-fact manner. Nevertheless, our analysis revealed that these early sexual experiences were characterized by clear power and age differences, and gender nonconformity contributed added vulnerability to CSA. Most did not disclose these encounters to their parents. Early sexual encounters often set the stage for further same-sex sexual encounters, and played a role in MSM sexual identity formation in some cases. Finally, CSA and transactional CSA may have also served as a potential gateway for sex work.

#### Power and age differences characterize early sexual experiences

All of these early sexual experiences entailed power and/or age differences, and participants rarely initiated these sexual encounters themselves. Participants described these experiences in different terms, ranging from clearly unwanted to partly or entirely desired and potentially enjoyable.

The majority of unwanted experiences involved older men in positions of authority, who approached participants when they were younger and the participant had little if any knowledge of sex. Family members, who had access to younger relatives, perpetrated instances of abuse at younger ages:

When I was 3 years old, my uncle did molestation, but I didn’t know that was sex and I didn’t know what he was doing. (Andhra Pradesh, Hyderabad, *DD*)

This abuse continued for another six or seven years until the participant’s family moved away. Several participants also reported abuse by teachers:

In the school, the teacher used to hug and kiss me. I did not know anything about this. (Karnataka, Tumkur, *kothi*)

Some of these cases involved sexual touching, while other cases also entailed anal sex.

I was in the 6th or the 7th standard [11–12 years old] at that time when all the other children went home he [the teacher] caught me and had [sex with me]. (Andhra Pradesh, Vijayawada, *kothi*)

In a few additional instances, however, the perpetrators of unwanted experiences were classmates and other peers who were closer in age to the participant:

When I used to go to school [age 10–12] the other boys used to take me and got me involved in homosexual activities. (Tamil Nadu, Madurai, *kothi*)

Even in this example, however, the “other boys” initiated the encounter, “tak[ing]” the participant and asserted their will.

Although participants sometimes mentioned elements of sexual desire, even these latter encounters were often linked to unwanted experiences. One participant’s description of such complex encounters is particularly revealing:

As far as I am concerned when I was the age of 6 to 7 years itself, I participated in sexual intercourse. It may be due to my interest also, it can be because I was girlish in appearance as a child and that interested and attracted guys I do not know whether I tried for them or they tried for me; from that time I am MSM. (Andhra Pradesh, Hyderabad, *kothi*)

Although the above participant expressed “interest” and attraction, children at ages of 6 or 7 cannot consent to sexual activities.

#### Gender nonconformity as an added vulnerability for CSA

Several participants, as the participant noted in the above example, mentioned that their gender nonconforming behavior played a role in these early sexual encounters. Some participants explicitly discussed that their gender nonconforming behavior made them vulnerable to being targeted by these men:

I used to play with the girls, talk to them and was behaving like girls. When I met someone I used to behave like a girl and I used to share my feeling with the girls and I felt like a girl from inside. One day the principal called me on pretext of giving tuition and had sex with me when he was drunk [age 14–15]. (Andhra Pradesh, Hyderabad, *kothi*)

In a similar example, a participant was systematically targeted by multiple men and other boys at school because of his gender nonconforming behavior:

I used to go to the pipe to take water and also for bath. There men used to come when they see me behaving like a girl they started giving me a chocolate and satisfy their urge for sex. I did not realize what was happening. Some used to give me 1 rupee. As I grew up and when I was in 7th standard [age 12 or 13], children used to fall on me and have sex with me. (Karnataka, Mangalore, *kothi*)

In this case CSA also entailed a transactional element, which we discuss below.

#### Lack of disclosure of CSA

Participants rarely discussed CSA with their parents, other family members, or friends, usually because of concerns that they would find this upsetting or because of fear from the abuser. For instance, the participant who was abused by his uncle from age 3 decided not to tell his parents even years after the abuse had taken place:

I didn’t tell what happened to me. I am HIV positive also. Such situations if my family knows they will be worried. When they scold me [for having HIV], I will get angry and I want to say, you should have taken care when I was young and that fellow has spoiled my life and I feel very worried and I am alone and I get angry on my uncle. (Andhra Pradesh, Hyderabad, *DD)*

This participant directly linked his abuse to his later same-sex sexual activities and subsequent HIV infection. Similarly, a participant who was abused by his brother stated:

I was scared of my brother. My mother used to work in school and then she used to go to take tuitions and back home late. My brother was at home and I was scared of him and I used to do whatever he said, but when will I tell [what happened to me]. (Tamil Nadu, Chennai, *kothi*)

#### CSA as an initiation experience for further same-sex sexual partnerships

Participants often noted that their early sexual encounter marked the beginning of further same-sex sexual experiences, sometimes referred to as a “habit.” In other descriptions an unwanted initial sexual encounter seemed to spark an interest in these sexual activities:

I was in my 8th standard [age 13–14] then, one of my elder brother’s friend used to stay with us, one night, thinking that I was sleeping he did sex (between the thighs) with me as if I am a girl, this happened a couple of time while I pretended to sleep, after that I too started actively participating in it. (Andhra Pradesh, Visakhapatnam, *kothi*)

These childhood sexual experiences with older boys and men were often incorporated into narratives about becoming MSM.

I used to look like a small child they took me to a man who looked like and uncle who liked me. He gave me something to drink and raped me. I was spoilt and now transformed in to an MSM. (Andhra Pradesh, Hyderabad, *kothi*)

In the above case the participant identified the abuse as the origin point for his sexual identity, which he experienced as corrupting or being “spoilt”. Many participants struggled with this kind of interpretation, which caused considerable confusion and suffering as they described their adult sexuality. These descriptions often included experiences of stigmatization by others, efforts by parents to “correct” their sexuality as well as internalized negative perceptions of their own sexual attraction and identity.

#### CSA as potential gateway to sex work

CSA that involves a transactional element, as in the example of a child receiving chocolate and token money above, may facilitate some MSM’s entry into sex work.

Several participants noted that many MSM begin their involvement in sex work at an early age:

I have seen 8–10 years old children engaged in MSM work in hotspot area who takes money for sucking. He gets 100–150 daily; per contact people give Rs. 20-Rs. 40 (Delhi, Delhi, *kothi*)

Other participants similarly noted the presence of children at sex work sites. One participant offered himself as an example of being brought into sex work by other MSM:

I am a live example and the age I started was when I was 6 or 7 years. It used to be like there are many kids who are very cute and sweet and so the children who are very interested [some MSM] will make them sit on their laps and allow them to stroke them gently and when their emotions reach the level of which they are unable to control then they have sex [with the child]. (Andhra Pradesh, Hyderabad, *kothi*)

Many participants clearly disapproved of children’s presence at sex work sites and of this kind of exploitation of children.

### Quantitative results

#### Demographics and HIV-related behaviors and experiences

Of the 11,997 MSM recruited, 11,788 (98.3 %) answered the interview question on CSA and were included in the analyses. Median age was 25 (interquartile range [IQR]: 21–32) (Table 1). 45.0 % self-identified as *panthi*, 14.0 % as *kothi*, and 18.0 % as *double deckers* (*DD*s); 15.3 % self-identified as *bisexual*, 5.9 % as *MSM*, and 1.8 % as *gay*. About one-third (35.3 %) were currently married to a woman or living with a partner. Few had injected illicit drugs (1.0 %) and 15.5 % reported non-injection drug use in the prior 6 months; 34.4 % had evidence of at least harmful or hazardous alcohol use and 15.2 % were alcohol dependent. Half (50.4 %) of the respondents reported UAI with a man in the prior 6 months; 19.1 % reported a history of sex work, of which 85.1 % reported recent sex work. Men reported a median of eight male sexual partners (IQR: 3–24) and two female sexual partners (IQR: 1–6) in their lifetime. A tenth reported ever experiencing intimate partner violence. Unweighted sample characteristics are presented in Additional file 1: Table S1.

**Table 1:** Characteristics^a^ by childhood sexual abuse^b^ (CSA) among 11,788 MSM in India

N (%), median (IQR)	No CSA (*N* = 7999)	CSA (*N* = 3789)	*p*-value*	Total (*N* = 11,788)
Median age	25 (21–32)	27 (22–35)	<0.001	25 (21–32)
Sexual identity				
*Panthi*	3228 (51.0)	640 (24.3)	<0.001	3868 (45.0)
*Kothi*	1188 (9.1)	1576 (30.7)		2764 (14.0)
*Double Deckers (DD)*	1771 (16.1)	980 (24.7)		2751 (18.0)
*Gay*	131 (1.5)	63 (2.9)		194 (1.8)
*MSM*	535 (6.0)	180 (5.8)		715 (5.9)
*Bisexual*	1146 (16.3)	350 (11.6)		1496 (15.3)
Marital Status				
Never married	4693 (62.0)	2032 (53.4)	0.044	6725 (60.1)
Currently married/living with partner	3003 (33.8)	1547 (40.6)		4550 (35.3)
Widowed/divorced/other	303 (4.2)	210 (6.0)		513 (4.6)
Education				
Primary school or less	1866 (21.5)	746 (18.6)	0.085	2612 (20.9)
Secondary school	3420 (45.0)	1707 (46.5)		5127 (45.3)
High school and above	2713 (33.5)	1336 (34.9)		4049 (33.8)
Employment				
Monthly/weekly wages	3861 (49.2)	1932 (55.0)	0.001	5793 (50.5)
Daily/seasonal wages	2769 (29.4)	1398 (30.9)		4167 (29.7)
Unemployed	304 (4.2)	154 (3.3)		458 (4.0)
Other (student or retired)	1065 (17.2)	305 (10.8)		1370 (15.8)
Depression^d^	768 (8.8)	805 (18.2)	<0.001	1573 (10.9)
HIV infection	637 (6.0)	490 (10.6)	<0.001	1127 (7.0)
Recent HIV-related risk behaviors				
Unprotected anal sex in prior 6 months				
No	2493 (31.5)	947 (23.7)	<0.001	3440 (29.8)
Yes	4266 (49.2)	2276 (54.3)		6542 (50.4)
No anal sex in prior 6 months	1239 (19.2)	565 (22.0)		1804 (19.8)
Median number of male sexual partners in prior 6 months	2 (1–3)	2 (1–5)	<0.001	2 (1–3)
Median number of female sexual partners in prior 6 months	1 (0–2)	0 (0–1)	0.006	1 (0–2)
Alcohol use and dependence^c^				
None/mild	5050 (67.0)	2028 (60.7)	0.001	7078 (65.6)
Harmful/hazardous	1617 (18.9)	805 (20.4)		2422 (19.2)
Alcohol dependence	1332 (14.1)	956 (18.9)		2288 (15.2)
Injection drug use in prior 6 months	99 (0.8)	41 (0.9)	0.291	140 (0.8)
Non-injection drug use in prior 6 months	1941 (16.1)	709 (13.5)	0.574	2650 (15.5)
Sex work in prior 6 months	1438 (12.7)	1612 (28.5)	<0.001	3050 (16.3)
Lifetime HIV-related risk behaviors and experiences				
Median number of lifetime male sexual partners	6 (3–20)	16 (6–54)	<0.001	8 (3–24)
Median number of lifetime female sexual partners	3 (1–7)	1 (0–4)	0.134	2 (1–6)
History of sex work	1726 (15.2)	1820 (32.5)	<0.001	3546 (19.1)
Early sexual debut (before age 15)	1612 (20.4)	1368 (31.7)	<0.001	2980 (25.6)
History of injection drug use	108 (1.1)	43 (0.9)	0.040	151 (1.0)
Ever experienced intimate partner violence	389 (3.1)	1512 (33.8)	<0.001	1901 (10.0)

^a^: Percentages and medians (IQR) are presented as RDS-II weighted. Percentages are column percentages

^b^: CSA defined as unwanted sexual experiences such as touching or sexual intercourse (either oral or anal) before 16 years old

^c^: Measured using AUDIT (Saunders, J. B., Aasland, OG, Babor, TF, De la Fuente, JR, Grant, M. Development of the alcohol use disorders identification test (AUDIT). WHO collaborative project on early detection of persons with harmful alcohol consumption-II. 1993 *Addiction, 88*, 791–791)

^d^: Depression defined as a score of 10 or more on the PHQ-9 (Kroenke K, Spitzer RL, Williams JB. The PHQ-9. *Journal of General Internal Medicine.* 2001;16(9):606–613)

* From multi-level logistic regression model likelihood ratio test comparing model with characteristic to intercept-only model of CSA

Scores of *recent* behaviors ranged from 0 to 7 and scores for *lifetime* behaviors and experiences ranged from 0 to 5; no participants reported all 6 *lifetime* behaviors/experiences (Table 2). A smaller portion of MSM who experienced CSA compared with those who had not experienced CSA reported none of the *recent* (7.6 % v. 12.9 %) or *lifetime* (31.3 % v. 56.9 %) risk behaviors/experiences we examined (*p*-value < 0.001). In turn, a larger portion of those who experienced CSA had 3 or more *recent* (34.4 % v 24.1 %) or *lifetime* (17.6 % v. 3.4 %) risk behaviors/experiences compared to those who had not experienced CSA. Distribution of unweighted scores by CSA status provided in Additional file 1: Table S2.

**Table 2:** HIV risk scores by childhood sexual abuse^a^ (CSA)

	No CSA	CSA	*p*-value*	Total
Number of recent behaviors^b^, n (%^d^)				
0	841 (12.9)	210 (7.6)	<0.001	1051 (11.7)
1	1948 (28.8)	651 (22.6)		2599 (27.4)
2	2630 (34.2)	1154 (35.4)		3784 (34.5)
3	1643 (16.7)	922 (21.0)		2565 (17.7)
4	706 (5.8)	655 (10.6)		1361 (6.9)
5	194 (1.3)	174 (2.3)		368 (1.5)
6	34 (0.3)	22 (0.5)		56 (0.3)
7	3 (0.02)	1 (0.001)		4 (0.02)
Number of lifetime behaviors/experiences^c^, n (%^d^)				
0	3957 (56.9)	763 (31.4)	<0.001	4720 (51.2)
1	2435 (29.6)	959 (29.7)		3394 (29.6)
2	1149 (10.1)	925 (21.3)		2074 (12.6)
3	395 (2.9)	777 (13.6)		1172 (5.3)
4	61 (0.5)	346 (3.9)		407 (1.2)
5	2 (0.005)	19 (0.2)		21 (0.04)
6	0 (0)	0 (0)		0 (0)

^a^: CSA defined as unwanted sexual experiences such as touching or sexual intercourse (either oral or anal) before 16 years old

^b^: Recent behaviors include: unprotected anal intercourse, high number of male sexual partners (6 or more), high number of female sexual partners (3 or more), hazardous alcohol use, injection drug use, non-injection drug use, and sex work. With the exception of hazardous alcohol use, which is in the prior 2 weeks, all other behaviors are within the prior 6 months

^c^: Lifetime behaviors/experiences include: high number of male sexual partners (55 or more), high number of female sexual partners (20 or more), ever injection drug use, ever sex work, ever intimate partner violence, and early sexual debut (under 15 years old)

^d^: Percentages and medians (IQR) are presented as RDS-II weighted

* From multi-level logistic regression model likelihood ratio test comparing model with behavior scores to intercept-only model of CSA

#### Prevalence of CSA

Overall prevalence of CSA was 22.4 % (95 % confidence interval [CI]: 18.9–25.9 %). The prevalence of CSA differed by sexual identity; nearly half (49.2 %) of *kothis* and one-third (30.8 %) of *DDs* reported CSA, while 17.0 % of *bisexuals* and 12.1 % of *panthis* reported CSA (*p*-value <0.001). CSA also varied considerably by state/region (*p*-value = 0.005). Tamil Nadu had the highest prevalence of CSA (43.2 %) with the city of Coimbatore at 70.2 % (Fig. 1). The Central/North region had the lowest prevalence (11.6 %) and Andhra Pradesh and Karnataka had similar prevalences, 21.2 and 22.6 %, respectively. Nearly a third of those participants who reported CSA also reported a history of sex work (32.5 %), compared to 15.2 % of those who did not experience CSA (*p*-value <0.001) (Table 1). Men who experienced CSA had approximately 2.5 times more lifetime male sexual partners compared to those without CSA (median of 16 and 6 male sexual partners, respectively; *p*-value <0.001). Moreover, a third (33.8 %) of those with CSA reported experiencing IPV in contrast to only 3.1 % of those without CSA (*p*-value <0.001). HIV prevalence among those that experienced CSA was almost twice as high compared to those without CSA (10.6 and 6.0 %, respectively; *p*-value <0.001).

#### CSA-risk score associations

In univariable analysis, CSA was associated with a 22 % increase in the rate of *recent* HIV-related behaviors (RR = 1.22, 95 % CI: 1.14–1.30, *p*-value < 0.001) and a 2.4 times increase in the rate of *lifetime* HIV-related behaviors and experiences (RR = 2.37, 95 % CI: 2.03–2.76, *p*-value < 0.001) (Table 3). In multivariable analysis, CSA remained significantly associated with the rate of *recent* HIV-related behaviors (aRR = 1.21, 95 % CI: 1.14–1.28, *p*-value < 0.001), holding sexual identity constant. After adjustment for age and sexual identity, CSA also remained significantly associated with the rate of *lifetime* HIV-related behaviors and experiences, with 2.0 times higher rate (aRR = 2.04, 95 % CI: 1.75–2.38, *p*-value < 0.001) among those that experienced CSA compared to those without CSA. Unweighted poisson model results for the HIV risk scores are presented in Additional file 1: Table S3. Univariable multilevel logistic regression results for each individual risk behavior/experience are presented in Additional file 1: Table S4.

**Table 3:** The relationship between CSA and number of HIV-risk behaviors and experiences^a^

	Recent score^b^						Lifetime score^c^					
	RR	95 % CI	*p*-value	aRR^d^	95 % CI	*p*-value	RR	95 % CI	*p*-value	aRR^e^	95 % CI	*p*-value
CSA	1.22	1.14–1.30	<0.001	1.21	1.14–1.28	<0.001	2.37	2.03–2.76	<0.001	2.04	1.75–2.38	<0.001
Age (by 5 years)	1.00	0.98–1.03	0.789	--	--		1.09	1.06–1.12	<0.001	1.06	1.03–1.08	<0.001
Sexual identity												
*Panthi*	REF			REF			REF			REF		
*Kothi*	1.07	0.93–1.24	0.343	1.10	0.93–1.09	0.939	2.34	2.04–2.69	<0.001	1.77	1.60–1.96	<0.001
*Double Deckers (DD)*	1.13	1.03–1.24	0.012	1.14	1.02–1.27	0.035	1.52	1.25–1.84	<0.001	1.32	1.12–1.54	0.001
*Gay*	0.96	0.81–1.14	0.667	0.90	0.75–1.07	0.342	1.14	0.86–1.51	0.354	0.99	0.78–1.26	0.925
*MSM*	1.10	0.72–1.67	0.652	1.02	0.69–1.50	0.661	1.08	0.74–1.57	0.688	1.07	0.76–1.49	0.697
*Bisexual*	1.11	0.99–1.23	0.062	1.14	1.04–1.24	0.018	1.28	1.07–1.53	0.007	1.19	0.99–1.43	0.057

^a^: With scaled RDS-II weights

^b^: Recent behaviors include: unprotected anal intercourse, high number of male sexual partners (6 or more), high number of female sexual partners (3 or more), hazardous alcohol use, injection drug use, non-injection drug use, and sex work. With the exception of hazardous alcohol use, which is in the prior 2 weeks, all other behaviors are within the prior 6 months

^c^: Lifetime behaviors/experiences include: high number of male sexual partners (55 or more), high number of female sexual partners (20 or more), ever injection drug use, ever sex work, ever intimate partner violence, and early sexual debut (under 15 years old)

^d^: Adjusted for sexual identity

^e^: Adjusted for sexual identity and age

*RR* rate ratio, *aRR* adjusted rate ratio

#### Sensitivity analysis

Since knowledge of an HIV-positive status could change the likelihood of engaging in risky sexual behaviors, we ran an additional multivariable model of *recent* risk behaviors excluding men who were aware of their HIV infection (*n* = 497). The effect of CSA on the rate of *recent* HIV-related behaviors excluding those aware of their HIV infection was very similar to that found in the full sample (aRR = 1.25, 95 % CI: 1.15–1.36, *p*-value < 0.001).

## Discussion

Our study of 11,788 MSM across 12 sites in India suggests that nearly a quarter (22.4 %) experienced CSA. This estimate is similar to the 23 % of boys who experienced more severe forms of CSA – the closest corresponding category of CSA comparable to our study – in the largest study of CSA in the general population of India to date [15]. Additionally, we found substantial differences in the prevalence of CSA across geographic regions and among specific sexual identities.

The geographic distribution of the prevalence of CSA among MSM in our study formed a gradient from North to South, with Tamil Nadu having a much higher prevalence of CSA than other states. Although only four of the states overlapped between the two studies (Andhra Pradesh, Delhi, Madhya Pradesh, and Uttar Pradesh), it is notable that the 2007 study found the highest prevalence of severe CSA among boys in Assam in the Northeast (65.6 %), and the second highest prevalence in Delhi (54.7 %), compared with a much lower prevalence of 8.8 % in Delhi in our study. Tamil Nadu was not included in the 2007 study, but an earlier study by Save the Children, which surveyed 2211 school children in Chennai in Tamil Nadu found that 48 % of boys experienced CSA there, indicating a high vulnerability of boys in the general population [15]. Our study used a definition of CSA restricted to unwanted sexual touching and intercourse prior to the age of 16, while the 2007 study employed a somewhat broader definition for severe CSA prior to the age of 18, and approached children directly instead of relying on self-reported recall in adulthood. These broader definitions would have likely yielded somewhat higher estimates for MSM than the general population reflected in the 2007 study. Additional comparative data using the same CSA assessment is necessary to determine whether MSM are at greater risk for being the victims of CSA compared to the general population in India.

The prevalence of CSA also substantially differed among different sexual identities, with nearly half of *kothis* and a third of *DDs* reporting CSA, while the prevalence of CSA was far lower among *bisexuals* and *panthis*. Although our qualitative sample included a much greater proportion of *kothis* than our quantitative sample, it was nevertheless striking that nearly all reports of CSA came from *kothis*. These findings align with the qualitative findings of Mimiaga and colleagues from Chennai, who found that *kothis* were particularly vulnerable to CSA [19]. Additionally, our qualitative data indicate that *kothis*’ often manifested gender nonconforming appearance and behavior during childhood and adolescence, which made them vulnerable to being targeted by older boys and men. This finding may explain *kothis’* disproportionately high prevalence of CSA. Our qualitative data yielded far fewer instances of CSA experiences from *DDs*, but *DD*s may also possess feminine characteristics during childhood and adolescence, which may make them comparatively more vulnerable to CSA than *bisexuals* and *panthis* who are less likely to manifest these characteristics. Our findings are consistent with data from the U.S. indicating that gender nonconforming boys may be at greater risk for CSA [58].

Although we did not investigate who the perpetrators were in our quantitative study, our qualitative findings suggest that many of perpetrators are older men and boys in positions of authority and power over children, including teachers and older relatives, whom children are expected to trust. This finding is consistent with previous research on CSA in India and elsewhere [13, 15, 19]. Such power dynamics make it very difficult for children to be able to escape an abusive situation and to report the abuse to their parents.

Qualitative findings suggest that our survey results may have captured a conservative estimate of the prevalence of CSA. The survey question restricts CSA to sexual experiences that are explicitly considered “unwanted” by the participant. Qualitative descriptions, however, suggested that participants may not always describe sexual encounters prior to 16 as strictly “unwanted,” even when they encompass unequal power dynamics and exploitation consistent with CSA [13], such as contact initiated by older boys and men when the child is not yet aware of or has limited knowledge of sex, using money or goods to entice a child, and encounters that led to an ongoing sexual relationship in which the participant actively engaged. Future research that addresses CSA among MSM in India should explore these more complex early sexual encounters to provide a more comprehensive portrait of the scope of CSA in this population.

Qualitative results clearly indicate that many MSM who experience CSA do not recognize the characteristic features of abuse, such as the unequal power dynamics described above, even in adulthood and may attribute the abuse to aspects of their own behavior. For instance, being targeted because of gender nonconformity may contribute to a sense that they are responsible for the abuse. The theme of self-stigmatization and self-blame is common among CSA survivors across cultural settings, and is detrimental to mental health and wellbeing [1]. Finally, participants also often link abuse to becoming an MSM, their sexual identity, and to their participation in sex work. This may enhance suffering and mental distress as participants negotiate their sexuality and cultural gender roles in a setting where homosexuality remains illegal and is highly stigmatized. This is an important area for future research and interventions that aim to educate children and families about CSA in order to reduce its prevalence, and that establish services to provide mental health care and respectful support for adult MSM struggling with aspects of their sexuality who have experienced CSA.

Our quantitative findings suggest that CSA had a substantial impact on the number of *recent* and *lifetime* sexual behaviors that place these men at elevated risk for HIV. The rate of *recent* HIV-related risk behaviors was 21 % higher among those who experienced CSA compared with those who did not report history of CSA. The impact of CSA was even more profound on *lifetime* HIV-related behaviors/experiences; those who experienced CSA had two times higher rate of *lifetime* HIV-related behaviors or experiences compared to MSM who did not experience CSA. Although we did not systematically investigate participants’ perspectives on the sequelae of CSA, qualitative research participants often discussed CSA as having initiated some high-risk sexual behaviors, including sexual activity with multiple male partners, and as an entry-point into sex work. Since our quantitative study was cross-sectional in nature, we could not assess the impact of CSA on HIV seroconversion over time. However, those who experienced CSA had 10.6 % prevalence of HIV compared with 6.0 % of those who did not report CSA. This difference in HIV prevalence is consistent with our findings of the differences in risk behaviors among MSM with and without histories of CSA.

Finally, while our paper focuses on the tremendous impact of CSA on HIV-related risk behaviors, it is important to note that some participants who experienced CSA did not go on to report either *recent* (7.6 %) or *lifetime* (31.4 %) HIV-related risk behaviors, respectively. There were also substantial differences in the prevalence of MSM who reported only one risk behavior compared to more than one. It is possible that this reflects differences in the different kinds of abuse that MSM experienced. In a recent study Boroughs and colleagues [59] found that there were substantial differences in the long-term psychological sequelae of abuse depending on the kind of abuse suffered, the relationship with the perpetrator, and the degree to which abuse persisted over time versus a single instance of abuse. Moreover, some participants may have psychological and social resources that make them more resilient in the face of abuse. Investigating these would be helpful in designing future interventions that address CSA in this population.

### Limitations

Our prevalence estimates may be influenced by our RDS methodology. Although adjustment for data collected via RDS attempts to produce valid population estimates, we cannot verify that our sample is representative of the underlying population and its associated characteristics. RDS methodology, however, was effective in recruiting a very diverse sample of MSM compared to other studies of psychosocial health and HIV risk behaviors among MSM that have relied on convenience or venue-based sampling. Our study is limited by its cross-sectional nature that does not allow us to examine the effects of CSA on HIV risk behaviors and HIV seroconversion over time. This limitation is mitigated by the fact that CSA took place prior to the age of 16, and all participants were adults 18 years or older at the time of the study. Not all variables of interest were available for both *lifetime* and *recent* timeframes. Future studies that investigate CSA could build on our insights and benefit from the inclusion of both sets of timeframes for all potentially relevant variables.

Our results on CSA are limited by the use of a single survey question that did not enable further elaboration of the context, perpetrator, and extent of CSA. Based on our qualitative findings, the instrument may have also underestimated CSA by addressing only explicitly “unwanted” sexual experiences under the age of 16. When children are surveyed directly about specific behaviors, they may also report more experiences of CSA, compared with when adults are asked to recollect these experiences, often after many years have passed. Nevertheless, the overall prevalence of CSA among MSM is quite high even according to these conservative estimates, and extremely high in the South and among *kothis*. Consequently, the combined qualitative and quantitative findings warrant further, more in-depth investigation of CSA in this population, as well as in comparison with the general population.

Our survey did not provide information about the causes of geographic variation for CSA, and our qualitative study could not offer any additional insights since CSA was an emergent theme rather than a topic that was an a priori topic of special interest. The underlying causes of this variation should be a topic of further investigations of CSA among MSM in India. Furthermore, although qualitative analyses yielded potential explanations for the greater vulnerabilities of *kothis* to CSA, we have far less understanding of the vulnerabilities of *DDs* and there is a clear need for additional investigation of the vulnerability of both of these sexual identities to CSA compared with other subgroups of MSM. Finally, our study does not address the resilience of those MSM who reported a history of CSA but did not go on to have any of the HIV-related risk factors that were included in our risk scores, which would benefit future interventions.

## Conclusion

This large, multi-site mixed methods study established an overall prevalence of 22.4 % of CSA among MSM, with substantially higher prevalence among MSM residing in the South and among more feminine sexual identities. Moreover, CSA was associated with a substantially elevated rate of *recent*, and an even higher rate of *lifetime* HIV-related risk factors. Future HIV-prevention efforts should survey the history of CSA among MSM and include mental health services that explicitly address the sequelae of CSA and are sensitive to the needs of MSM who have diverse social and sexual identities, sexual orientations and gender expression. Moreover, additional in-depth studies of CSA among both MSM and the general population are needed in order to develop effective CSA interventions that are sensitive to the diversity of vulnerabilities of children and adolescents as they form their own sexual identities.

## Abbreviations

CSA, childhood sexual abuse; DD, double decker; FGD, focus group discussion; IDI, in-depth interview; LMIC, low and middle income countries; MSM, men who have sex with men; PTSD, posttraumatic stress disorder; STI, sexually transmitted infection; UAI, unprotected anal intercourse; YRGCARE, YR Gaitonde Centre for AIDS Research and Education

## Additional file

Additional file 1: Table S1. Unweighted characteristics by childhood sexual abuse^1^ (CSA) among 11,788 men who have sex with men in 12 Indian cities. **Table S2.** Number of HIV risk behaviors and experiences by childhood sexual abuse^1^ (CSA). **Table S3.** The relationship between CSA and HIV-related risk score. **Table S4.** The relationship between CSA and recent and lifetime HIV-related risk behaviors and experiences^1^. (DOCX 37 kb)
