# Supplementary material for: The prevalence and impact of childhood sexual abuse on HIV-risk behaviors among men who have sex with men (MSM) in India
Source: BMC Public Health. 2016 Aug 12;16:784. doi: 10.1186/s12889-016-3446-6 (PMC4983056; doi:10.1186/s12889-016-3446-6)
Supplement: Additional file 1: Table S1. — Unweighted characteristics by childhood sexual abuse1 (CSA) among 11,788 men who have sex with men in 12 Indian cities. Table S2. Number of HIV risk behaviors and experiences by childhood sexual abuse1 (CSA). Table S3. The relationship between CSA and HIV-related risk score. Table S4. The relationship between CSA and recent and lifetime HIV-related risk behaviors and experiences1. (DOCX 37 kb) [file 12889_2016_3446_MOESM1_ESM.docx]

**Additional file 1: Table S1: Unweighted characteristics by childhood sexual abuse^1^ (CSA) among 11,788 men who have sex with men in 12 Indian cities**

| N (%), median (IQR) | No CSA (N=7,999) | CSA  (N=3,789) | p-value* | Total (N=11,788) |
| --- | --- | --- | --- | --- |
| Site (row %)  Bengaluru  Belgaum  Bhopal  Chennai  Coimbature  New Delhi  Hyderabad  Lucknow  Madurai  Mangalore  Vijayawada  Visakhapatnam | 734 (75.4)  757 (81.5)  840 (85.1)  611 (61.4)  281 (28.4)  815 (82.0)  601 (60.3)  801 (80.2)  554 (55.6)  648 (65.5)  797 (80.6)  560 (59.0) | 240 (24.6)  172 (18.5)  147 (14.9)  384 (38.6)  707 (71.6)  179 (18.0)  396 (39.7)  198 (19.9)  443 (44.4)  341 (34.5)  192 (19.4)  390 (41.1) | <0.001 | 974  929  987  995  988  994  997  999  997  989  989  950 |
| Region/State (row %)  Andhra Pradesh  Karnataka  Tamil Nadu  Central/North | 1958 (66.7)  2139 (74.0)  1446 (48.5)  2456 (82.4) | 978 (33.3)  753 (26.0)  1534 (51.5)  524 (17.6) | <0.001 | 2936  2892  2980  2980 |
| Median age | 26 (21 – 33) | 27 (22 – 35) | <0.001 | 26 (21 – 33) |
| Sexual identity  *Panthi*  *Kothi*  *Double Deckers (DD)*  *Gay*  *MSM*  *Bisexual* | 3228 (40.4)  1188 (14.9)  1771 (22.1)  131 (1.6)  535 (6.7)  1146 (14.3) | 640 (16.9)  1576 (41.6)  980 (25.9)  63 (1.7)  180 (4.8)  350 (9.2) | <0.001 | 3868 (32.8)  2764 (23.5)  2751 (23.3)  194 (1.7)  715 (6.1)  1496 (12.7) |
| Marital Status  Never married  Currently married/living with partner  Widowed/divorced/other | 4693 (58.7)  3003 (37.5)  303 (3.8) | 2032 (53.6)  1547 (40.8)  210 (5.5) | <0.001 | 6725 (57.1)  4550 (38.6)  513 (4.4) |
| Education  Primary school or less  Secondary school  High school and above | 1866 (23.3)  3420 (42.8)  2713 (33.9) | 746 (19.7)  1707 (45.1)  1336 (35.3) | <0.001 | 2612 (22.2)  5127 (43.5)  4049 (34.4) |
| Employment  Monthly/weekly wages  Daily/seasonal wages  Unemployed  Other (student or retired) | 3861 (48.3)  2769 (34.6)  304 (3.8)  1065 (13.3) | 1932 (51.0)  1398 (36.9)  154 (4.1)  305 (8.1) | <0.001 | 5793 (49.1)  4167 (35.4)  458 (3.9)  1370 (11.6) |
| Depression^3^ | 768 (9.6) | 805 (21.3) | <0.001 | 1573 (13.3) |
| HIV infection | 637 (8.0) | 490 (12.9) | <0.001 | 1127 (9.6) |
| Recent HIV-related risk behaviors | | | | |
| Unprotected anal sex in prior 6 months  No  Yes  No anal sex in prior 6 months | 2493 (31.2)  4266 (53.3)  1239 (15.5) | 947 (25.0)  2276 (60.1)  565 (14.9) | <0.001 | 3440 (29.2)  6542 (55.5)  1804 (15.3) |
| Median number of male sexual partners in prior 6 months | 2 (1 - 5) | 3 (1 - 13) | <0.001 | 2 (1 - 6) |
| Median number of female sexual partners in prior 6 months | 1 (0-2) | 0 (0-1) | <0.001 | 1 (0-1) |
| Alcohol use and dependence^2^  None/mild  Harmful/hazardous  Alcohol dependence | 5050 (63.1)  1617 (20.2)  1332 (16.7) | 2028 (53.5)  805 (21.3)  956 (25.2) | <0.001 | 7078 (60.0)  2422 (20.6)  2288 (19.4) |
| Injection drug use in prior 6 months | 99 (1.2) | 41 (1.1) | 0.465 | 140 (1.2) |
| Non-injection drug use in prior 6 months | 1941 (24.3) | 709 (18.7) | <0.001 | 2650 (22.5) |
| Sex work in prior 6 months | 1438 (18.0) | 1612 (42.6) | <0.001 | 3050 (25.9) |
| Lifetime HIV-related risk behaviors and experiences | | | | |
| Median number of lifetime male sexual partners | 10 (4 - 35) | 40 (10 - 200) | <0.001 | 15 (5 - 55) |
| Median number of lifetime female sexual partners | 2 (1-7) | 1 (0-4) | <0.001 | 2 (0-6) |
| History of sex work | 1726 (21.7) | 1820 (48.1) | <0.001 | 3546 (30.2) |
| Early sexual debut (before age 15) | 1612 (20.4) | 1368 (36.7) | <0.001 | 2980 (25.6) |
| History of injection drug use | 108 (1.4) | 43 (1.1) | 0.332 | 151 (1.3) |
| Ever experienced intimate partner violence | 389 (4.9) | 1512 (40.0) | <0.001 | 1901 (16.1) |

1: CSA defined as unwanted sexual experiences such as touching or sexual intercourse (either oral or anal) before 16 years old.

*P-value from Person chi-square test for categorical characteristics and Wilcoxon rank sum for continuous characteristics.

Percentages are column percentages, unless otherwise noted.

2: Measured using AUDIT (Saunders, J. B., Aasland, OG, Babor, TF, De la Fuente, JR, Grant, M. Development of the alcohol use disorders identification test (AUDIT). WHO collaborative project on early detection of persons with harmful alcohol consumption-II. 1993 *Addiction, 88*, 791-791)

3: Depression defined as a score of 10 or more on the PHQ-9 (Kroenke K, Spitzer RL, Williams JB. The PHQ-9. *Journal of General Internal Medicine.* 2001;16(9):606-613)

**Additional file 1: Table S2: Number of HIV risk behaviors and experiences by childhood sexual abuse^1^ (CSA)**

|  | No CSA | CSA | p-value* | Total |
| --- | --- | --- | --- | --- |
| Number of recent behaviors^2^, n (%)  0  1  2  3  4  5  6  7 | 841 (10.5)  1948 (24.4)  2630 (32.9)  1643 (20.5)  706 (8.8)  194 (2.4)  34 (0.4)  3 (0.04) | 210 (5.5)  651 (17.2)  1154 (30.5)  922 (24.3)  655 (17.3)  174 (4.6)  22 (0.6)  1 (0.03) | <0.001 | 1051 (8.9)  2599 (22.1)  3784 (32.1)  2565 (21.8)  1361 (11.6)  368 (3.1)  56 (0.5)  4 (0.03) |
| Number of lifetime behaviors/experiences^3^, n (%)  0  1  2  3  4  5  6 | 3957 (49.5)  2435 (30.4)  1149 (14.4)  395 (4.9)  61 (0.8)  2 (0.03)  0 (0) | 763 (20.1)  959 (25.3)  925 (24.4)  777 (20.5)  346 (9.1)  19 (0.5)  0 (0) | <0.001 | 4720 (40.0)  3394 (28.8)  2074 (17.6)  1172 (9.9)  407 (3.5)  21 (0.2)  0 (0) |

1: CSA defined as unwanted sexual experiences such as touching or sexual intercourse (either oral or anal) before 16 years old.

2: Recent behaviors include: unprotected anal intercourse, high number of male sexual partners (6 or more), high number of female sexual partners (3 or more), hazardous alcohol use, injection drug use, non-injection drug use, and sex work. With the exception of hazardous alcohol use, which is in the prior 2 weeks, all other behaviors are within the prior 6 months.

3: Lifetime behaviors/experiences include: high number of male sexual partners (55 or more), high number of female sexual partners (20 or more), ever injection drug use, ever sex work, ever intimate partner violence, and early sexual debut (under 15 years old).

*P-value from Person chi-square test

**Additional file 1: Table S3: The relationship between CSA and HIV-related risk score**

|  | Recent score^1^ | | | | | | Lifetime score^2^ | | | | | |
| --- | --- | --- | --- | --- | --- | --- | --- | --- | --- | --- | --- | --- |
|  | **RR** | **95% CI** | **p-value** | **aRR^3^** | **95% CI** | **p-value** | **RR** | **95% CI** | **p-value** | **aRR^3^** | **95% CI** | **p-value** |
| CSA | 1.23 | 1.19 - 1.27 | <0.001 | 1.22 | 1.18 - 1.26 | <0.001 | 2.25 | 2.17 - 2.34 | <0.001 | 1.90 | 1.82 - 1.97 | <0.001 |
| Age (by 5 years) | 1.00 | 0.99 - 1.01 | 0.860 | -- | -- |  | 1.07 | 1.06 - 1.08 | <0.001 | 1.10 | 1.07 - 1.12 | <0.001 |
| Sexual identity  *Panthi*  *Kothi*  *Double Deckers (DD)*  *Gay*  *MSM*  *Bisexual* | REF  1.11  1.12  0.99  1.19  1.10 | 1.07 - 1.15  1.08 - 1.16  0.88 - 1.12  1.12 - 1.27  1.05 - 1.15 | <0.001  <0.001  0.923  <0.001  <0.001 | REF  1.03  1.14  0.91  1.07  1.14 | 0.99 - 1.08  1.09 - 1.18  0.80 - 1.03  1.00 - 1.15  1.09 - 1.20 | 0.472  <0.001  0.4400  <0.001  0.002 | REF  2.40  1.56  1.23  1.06  1.26 | 2.29 - 2.51  1.48 - 1.65  1.05 - 1.45  0.95 - 1.17  1.18 - 1.35 | <0.001  <0.001  0.011  0.308  <0.001 | REF  1.81  1.35  1.11  1.03  1.16 | 1.72 - 1.91  1.28 - 1.42  0.94 - 1.30  0.93 - 1.14  1.08 - 1.24 | <0.001  <0.001  0.226  0.579  <0.001 |

1: Recent behaviors include: unprotected anal intercourse, high number of male sexual partners (6 or more), high number of female sexual partners (3 or more), hazardous alcohol use, injection drug use, non-injection drug use, and sex work. With the exception of hazardous alcohol use, which is in the prior 2 weeks, all other behaviors are within the prior 6 months.

2: Lifetime behaviors/experiences include: high number of male sexual partners (55 or more), high number of female sexual partners (20 or more), ever injection drug use, ever sex work, ever intimate partner violence, and early sexual debut (under 15 years old).

3: Adjusted for sexual identity

4: Adjusted for sexual identity and age

RR: rate ratio; aRR: adjusted rate ratio

**Additional file 1: Table S4: The relationship between CSA and recent and lifetime HIV-related risk behaviors and experiences^1^**

|  | Unweighted Odds Ratio | 95% CI | p-value | Weighted Odds Ratio^1^ | 95% CI | p-value |
| --- | --- | --- | --- | --- | --- | --- |
| Recent behaviors | | | | | | |
| Unprotected anal sex in prior 6 months | 1.41 | 1.29 - 1.53 | <0.001 | 1.42 | 1.31 - 1.54 | <0.001 |
| High number of male sexual partners in prior 6 months (6 or more) | 2.72 | 2.46 - 3.01 | <0.001 | 2.73 | 1.93 - 3.86 | <0.001 |
| High number of female sexual partners in prior 6 months (3 or more) | 0.70 | 0.62 - 0.80 | <0.001 | 0.95 | 0.58 - 1.54 | 0.828 |
| Hazardous alcohol use | 1.23 | 1.13 - 1.34 | <0.001 | 1.18 | 0.97 - 1.45 | 0.105 |
| Injection drug use in prior 6 months | 0.92 | 0.63 - 1.36 | 0.679 | 0.78 | 0.33 - 1.84 | 0.565 |
| Non injection drug use in prior 6 months | 0.90 | 0.81 - 1.00 | 0.054 | 0.97 | 0.64 - 1.45 | 0.867 |
| Sex work in prior 6 months | 3.29 | 3.00 - 3.62 | <0.001 | 2.69 | 2.08 - 3.48 | <0.001 |
| Lifetime behaviors and experiences | | | | | | |
| High number of lifetime male sexual partners (55 or more) | 3.45 | 3.12 - 3.83 | <0.001 | 3.48 | 2.41 - 5.01 | <0.001 |
| High number of lifetime female sexual partners (20 or more) | 0.69 | 0.60 - 0.79 | <0.001 | 0.84 | 0.57 - 1.24 | <0.001 |
| Ever injection drug use | 0.88 | 0.60 - 1.28 | 0.496 | 0.63 | 0.28 - 1.43 | 0.268 |
| Ever sex work | 3.29 | 3.00 - 3.61 | <0.001 | 2.62 | 1.98 - 3.46 | <0.001 |
| Ever intimate partner violence | 12.27 | 10.79 - 13.96 | <0.001 | 13.62 | 8.39 - 22.12 | <0.001 |
| Early sexual debut (under 15 years old) | 3.89 | 3.51 - 4.31 | <0.001 | 3.47 | 2.71 - 4.46 | <0.001 |

1: With scaled RDS-II weights.
